# Defamation Against Healthcare Workers During COVID-19 Pandemic

**DOI:** 10.34172/ijhpm.2020.184

**Published:** 2020-09-27

**Authors:** Kazuki Shimizu, Leesa Lin

**Affiliations:** ^1^Authors’ affiliations 1 Department of Health Policy, London School of Economics and Political Science, London, UK.; ^2^Faculty of Public Health and Policy, London School of Hygiene and Tropical Medicine, London, UK.; ^3^Graduate School of Medicine, Hokkaido University, Sapporo, Japan.; ^4^Department of Infectious Disease Epidemiology, London School of Hygiene and Tropical Medicine, London, UK.

## Dear Editor,

 While front-line healthcare workers (HCWs) combatting coronavirus disease 2019 (COVID-19) have commanded much respect and praise,^1^ they have been persecuted in Japan – a collective society where individual reputation and work ethic are traditionally viewed as important as one’s life. HCWs, who were exposed to higher risk of infection due to lack of sufficient personal protective equipment and mentally exhausted in daily work, have another epidemic to fight: defamation. They have been targeted for discrimination and abuse in off-duty hours and stigmatized as “germs” in public spaces with many suffering social sanctions in forms of social exclusion and even bullying.^2^

 Anxiety and fear among Japanese citizens were generated in January,^3^ and driven by the increase of nosocomial infections in February-April.^4^ Healthcare facilities and HCWs were seen as epicenters, which triggered widespread irrational prejudice and discrimination against HCWs. They were denied use of public buses, taxis, and even urged to vacate rental housing. Family members also suffered from defamation. Children of HCWs were asked to voluntarily refrain from using nursery schools or discriminated as “You are COVID-19” in their schools.^2^ Concurrently, there has been peer pressure among HCWs to commit to working on the front-line, fueled by the strong sense of responsibility that runs deep in Japanese work culture. HCWs had to struggle with the tension between the burnout and social sanctions, resulting in a wave of temporary or permanent leaves of absence^2^ and consequently damaging the country’s health system capacity.

 To understand the discrimination against HCWs in Japan, root causes of the public anxiety towards COVID-19 must be investigated. First, public panic was incited by the interrupted access to testing.^5^ Due to the lack of testing capacity, only a small portion of the infected cases has been identified,^6-8^ leading to public panic towards a largely underdiagnosed population. Unmet needs of health services are unprecedented and unfamiliar to Japanese residents who have historically enjoyed the resilient health system that ensured free access to healthcare.^9,10^ Second, while designated medical institutions for infectious diseases continued accepting COVID-19 patients, some hospitals avoided them. In Tokyo, the number of rejection of ambulance cars amounted to 2365 in April 2020, which was nearly quadrupled compared to April 2019.^11^ Third, there has been a lack of governmental leadership to support HCWs; in fact, Japanese government’s emergency risk communication was identified as a weakness that has negatively impacted its response to COVID-19.^5^ To date, there has been scant strong and clear messaging from political leaders that shows solidarity with front-line HCWs or any public acknowledgement of their commitment to protecting people’s health during the pandemic. Fourth, there is a lack of strong medical journalism in Japan. Sensational media reports that are not based on scientific evidence have sometimes disseminated false information to the public.^12^ Fifth, Japan’s declaration of State of Emergency, which was made between April and May, is not enforceable by law. All countermeasures such as remote working and staying indoors were on a voluntary basis. The strong peer pressure among Japanese has worked as a double-edged sword, which not only positively worked for infection control but negatively made Japanese residents suspicious of each other. Vigilantes have emerged, and coronavirus harassment for those who do not confirm has further divided the society.^13^ HCWs were socially excluded by an agitated public and irrationally criticized as the source of COVID-19.

 Even before the COVID-19 pandemic, the pressure of deep-rooted conformity and social sanction that intersected with public health threats in Japanese society have long been documented. In 2011, stigmatized evacuees of the Fukushima Daiichi nuclear disaster were bullied at school,^14^ highlighting an urgent need for context-specific public health education tailored to the Japanese sociocultural environment. During the 2009 influenza A (H1N1) pandemic, discrimination against front-line HCWs was acknowledged as a critical public health crisis.^15^ It has been a decade since Japan was tested with a pandemic, yet not much progress has been made despite the evidence that the feeling of being supported by the government with frequent communication and encouragement will motivate HCWs.^15^

 To continue the fight against COVID-19, public leaders must ensure citizens’ access to testing, take responsibility of HCWs’ safety, show strong solidarity supporting HCWs, and prepare effective communication strategies tackling discrimination and social sanction against front-line HCWs.

## Ethical issues

 Not applicable.

## Competing interests

 Authors declare that they have no competing interests.

## Authors’ contributions

 KS conceived the presented data, collected, and analyzed the data. KS drafted the initial manuscript. LL critically reviewed and revised the manuscript. Both authors have reviewed, commented on, and approved the final version of the manuscript.

## Funding

 KS receives research support in the United Kingdom by The Rotary Foundation ‘[grant number: GG1986485];’ Japan Student Services Organization ‘[grant number: NM1910100304];’ and the British Council Japan Association. The funders had no role in study design, data collection and analysis, decision to publish, or preparation of the manuscript.

## Authors’ affiliations

^1^Department of Health Policy, London School of Economics and Political Science, London, UK. ^2^Faculty of Public Health and Policy, London School of Hygiene and Tropical Medicine, London, UK. ^3^Graduate School of Medicine, Hokkaido University, Sapporo, Japan. ^4^Department of Infectious Disease Epidemiology, London School of Hygiene and Tropical Medicine, London, UK.
